# Supplementary material for: Effect of Dietary Salt Intake on Risk of Gastric Cancer: A Systematic Review and Meta-Analysis of Case-Control Studies
Source: Nutrients. 2022 Oct 12;14(20):4260. doi: 10.3390/nu14204260 (PMC9609108; doi:10.3390/nu14204260)
Supplement: Supplementary file 1 [file nutrients-14-04260-s001.zip › nutrients-1889898-supplementary.pdf]

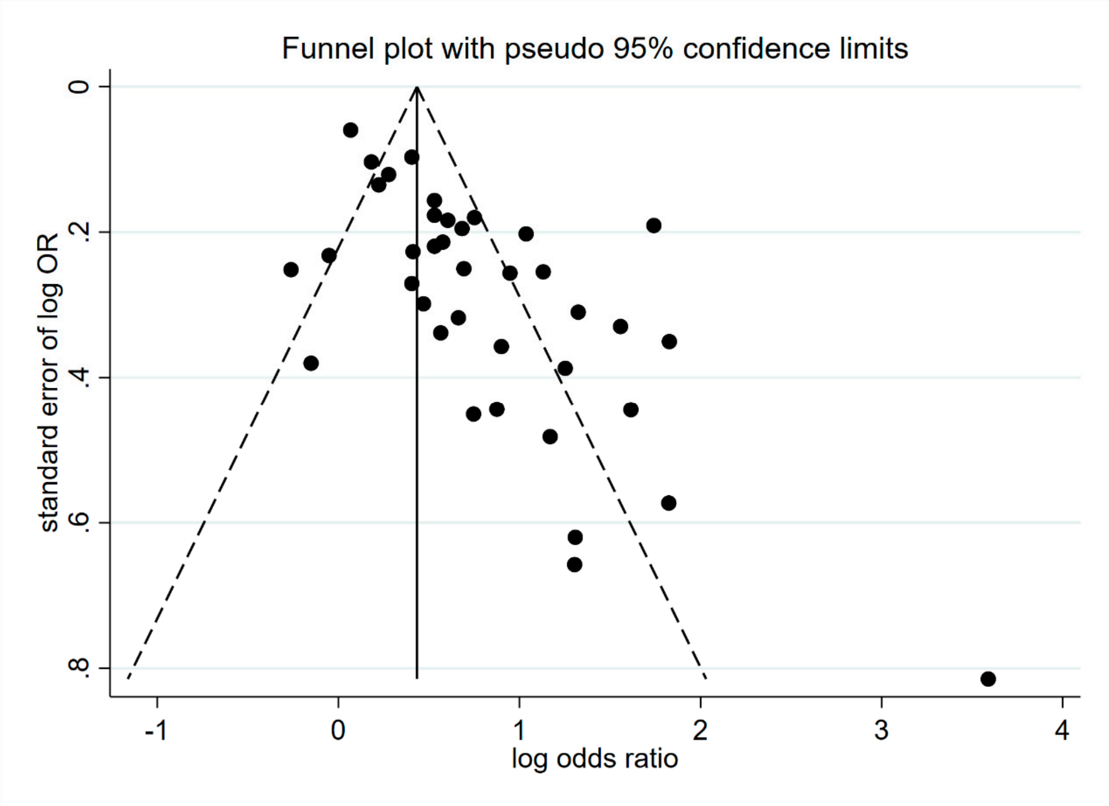

Figure S1. Funnel plots for identifying publication bias in the meta-analysis of observational studies.

Abbreviations: OR, odds ratio.

Table S1 The PRISMA checklist

Section and Topic	Item	Location where item is reported
**TITLE**		
Title	1	The report is identified as a systematic review and a meta-analysis.
**ABSTRACT**		
Abstract	2	The structured abstract includes Aim, Methods, Results and Conclusion.
**INTRODUCTION**		
Rationale	3	Described in the Introduction.
Objectives	4	Described in the Abstract and the Introduction.
**METHODS**		
Eligibility criteria	5	They are defined in the Methods.
Information sources	6	Described in the Methods.
Search strategy	7	Described in the Methods.
Selection process	8	Described in the Methods.
Data collection process	9	Described in the Methods.
Data items	10	Described in the Methods and summarized in Table 1 and Appendix Table s2.
Study risk of bias assessment	11	Assessed with Newcastle-Ottawa scale and described in the Methods. Shown in Table 1 and Appendix Table s3.
Effect measures	12	Odds Ratio.
Synthesis methods	13	Described in Statistical analysis and reported in detail in Results.
Reporting bias assessment	14	This have not provided.
Certainty assessment	15	This have not provided.
**RESULTS**		
Study selection	16	See Flow Diagram in Figure 1.
Study characteristics	17	Described in Table 1 and Appendix Table s2.
Risk of bias in studies	18	Assessed with Newcastle-Ottawa scale and described in Table 1 and Appendix Table s3.
Results of individual studies	19	Described in Results and shown in Figure 2, Figure 3, Figure 4 and Table 2.
Results of syntheses	20	Described in Results and shown in Figure 2, Figure 3 and Figure 4.
Reporting biases	21	Described in the Discussion.
Certainty of evidence	22	This have not provided.

Section and Topic	Item	Location where item is reported
**DISCUSSION**		
Discussion	23	All details described in the Discussion.
**OTHER INFORMATION**		
Registration and protocol	24	The protocol is described in the Methods. The meta-analysis has registered in PROSPERO website (https://www.crd.york.ac.uk/prospero/)(ID: CRD42022354245).
Support	25	This research was supported by the National Natural Science Foundation of China (grant number: 82273676) and the national key research and development program of China (grant numbers: 2021YFA1301200, 2021YFA1301202).
Competing interests	26	None.
Availability of data, code and other materials	27	The data that support the findings of this study are available on request from the corresponding authors.

Table S2. Adjustment variables of the case-control studies included in the meta-analysis.

First author	Publication year	Adjustment variables
Tuyns[20]	1988	age, sex, and province
Buiatti[21]	1989	age, sex, area, place of residence, migration from the south, socio-economic status, familial history of GC, Quetelet index, tertile levels of consumption of one or more dietary variables
Negri[22]	1990	not refer
Demirer[23]	1990	not refer
Hoshiyama[24]	1992	not refer
Ramón[25]	1993	sex, age, education, cigarettes/day, rice, citrus fruit, raw green vegetables, all fruits, cereals, smoked and pickled foods
Nazario[26]	1993	not refer
Hansson[27]	1993	age, gender, socio-economic status
Lee[28]	1995	age, sex, education, economic status, residence, other dietary factors
Vecchia[29]	1997	sex, age, education
Ye[15]	1998	not refer
Ji[30]	1998	age, sex, income, education, smoking, alcohol drinking
Ward[31]	1999	age, gender, total calories, chili pepper consumption, added salt, history of peptic ulcer, cigarette smoking, and socioeconomic status
Palli[32]	2001	age, sex, social class, family history of gastric cancer, area of rural residence, BMI tertiles, total energy, tertiles of the residuals of each nutrient of interest.
Sriamporn[33]	2002	age, sex, fermentated food
Kim[34]	2002	sex, age, socioeconomic status, family history and refrigerator use
Sun[35]	2002	age, income, resident space, using refrigerator and educational level
Lee[36]	2003	age, sex, education, family history of GC, smoking, drinking, *H. pylori* infection
Stefani[37]	2004	Age, sex, residence, urban/rural status, education, BMI, total energy intake
Lissowska[38]	2004	age, sex, education, smoking, calories from foods
Qiu[39]	2005	age, present residence, education, economic status, smoking, alcoholics, total calories intake
Campos[40]	2006	not refer
Hsu[41]	2008	carriage of myeloperoxidase allele A, gender, advanced age, tea consumption, level of education, *H. pylori* infection
Pelucchi[42]	2009	Age, sex, adjusted for period of interview, education, BMI, tobacco smoking, family history of stomach cancer, total energy intake
Pourfarzi[43]	2009	gender, age group, education, family history of GC, citrus fruits, garlic, onion, red, meat, fish, dairy products, strength and warmth of tea, preference for salt intake and *H. pylori*
Wen[44]	2010	age, sex, BMI, family history, smoking, drinking, fresh fruit, fresh vegetables
Peleteiro[16]	2011	gender, age, education, smoking and *H. pylori* infection
Yang[45]	2011	age, sex, smoking, drinking, fresh fruit and fresh vegetables;
Lazarević[46]	2011	not refer
Zhang[47]	2011	sex, age, education level, smoking, drinking, *H. pylori* infection
Hu[48]	2011	age group, province, education, BMI, sex, alcohol drinking, pack-years smoking, total vegetable and fruit intake, total energy intake;
Pakseresht[49]	2011	age, sex, education, living area, smoking, gastric symptoms, income, owning refrigerator, duration of using refrigerator, seeds preparing method, frying, *H. pylori* infection, total energy intake
Yassıbaş[50]	2012	gender, residence, education, smoking, alcohol consumption and family history of cancer for 26 kinds of foods considered to be related to gastric cancer
Chen [9]	2012	sex, age, education level, smoking, drinking, *H. pylori* infection
Epplein[51]	2014	age, smoking, history of gastritis, regular aspirin use, average, total energy intake
Lin[52]	2014	age, sex, home income, family history of cancer, smoking status, alcohol drinking, fresh vegetables intake, fresh fruit intake
Salvador[17]	2015	not refer
Kwak[18]	2021	Age, sex, BMI, education level, family history of gastric cancer, smoking status, alcohol drinkers, total energy intake, *H. pylori* infection

Abbreviations: GC, gastric cancer, *H. pylori*: Helicobacter pylori, BMI: body mass index.

Table S3 The study quality scores of the studies included in meta-analysis

First author	Selection				Comparability	Exposure		
	Is the case adequate definition	Representativeness of the cases	Selection of Controls	Definition of Controls	Comparability of cases and controls on the basis of the design or analysis	Ascertainment of exposure	Same method of ascertainment for cases and controls	Non-Response rate
Tuyns[20]	0	*	*	*	0	*	*	0
Buiatti[21]	*	*	*	*	0	*	*	0
Negri[22]	*	*	*	*	**	*	*	0
Demirer[23]	*	*	0	*	**	*	*	0
Hoshiyama[24]	*	*	*	*	0	*	*	0
Ramón[25]	*	*	*	*	**	*	*	0
Nazario[26]	*	*	*	*	**	*	*	0
Hansson[27]	*	*	*	*	**	*	*	0
Lee[28]	*	*	0	*	**	*	*	0
Vecchia[29]	*	*	0	*	0	*	*	0
Ye[15]	*	*	*	*	**	*	*	0
Ji[30]	*	*	*	*	**	*	*	0
Ward[31]	*	*	*	*	*	*	*	*
Palli[32]	*	*	*	*	0	*	*	0
Sriamporn[33]	*	*	0	*	**	*	*	0
Kim[34]	*	*	0	*	**	*	*	0
Sun[35]	*	*	*	*	**	*	*	0
Lee[36]	*	*	0	*	**	*	*	0
Stefani[37]	*	*	0	*	**	*	*	0
Lissowska[38]	*	*	*	*	**	*	*	0

Qiu[39]	*	*	*	*	0	*	*	0
Campos[40]	*	*	0	*	**	*	*	0
Hsu[41]	*	*	*	*	**	*	*	0
Pelucchi[42]	*	*	0	*	**	*	*	0
Pourfarzi[43]	*	*	*	*	**	*	*	0
Wen[44]	*	*	0	*	**	*	*	0
Peleteiro[16]	*	*	*	*	**	*	*	0
Yang[45]	*	*	0	*	**	*	*	0
Lazarević[46]	*	*	0	*	**	*	*	0
Zhang[47]	*	*	*	*	0	*	*	0
Hu[48]	*	*	*	*	0	*	*	0
Pakseresht[49]	*	*	*	*	0	*	*	0
Yassıbaş[50]	*	*	0	*	**	*	*	0
Chen [9]	*	*	*	*	0	*	*	0
Epplein[51]	*	*	*	*	**	*	*	0
Lin[52]	*	*	0	*	**	*	0	0
Salvador[17]	*	*	0	*	**	*	*	0
Kwak[18]	*	*	0	*	**	*	*	0
